# Exciton-Related Raman Scattering, Interband Absorption and Photoluminescence in Colloidal CdSe/CdS Core/Shell Quantum Dots Ensemble

**DOI:** 10.3390/nano11051274

**Published:** 2021-05-12

**Authors:** Grigor A. Mantashian, Paytsar A. Mantashyan, Hayk A. Sarkisyan, Eduard M. Kazaryan, Gabriel Bester, Sotirios Baskoutas, David B. Hayrapetyan

**Affiliations:** 1 Institute of Engineering and Physics, Russian-Armenian University, Yerevan 0051, Armenia; grigor.mantashyan@rau.am (G.A.M.); paytsar.mantashyan@rau.am (P.A.M.); hayk.sarkisyan@rau.am (H.A.S.); eduard.ghazaryan@rau.am (E.M.K.); 2 Institute for Physical Research of NAS RA, Ashtarak 0203, Armenia; 3 Institut für Physikalische Chemie, Universität Hamburg, Grindelallee 117, 20146 Hamburg, Germany; gabriel.bester@chemie.uni-hamburg.de (G.B.); bask@upatras.gr (S.B.); 4 Department of Materials Science, University of Patras, GR-26504 Patras, Greece

**Keywords:** raman scattering, exciton, interband absorption, luminescence, CdSe/CdS, core/shell quantum dot

## Abstract

By using the numerical discretization method within the effective-mass approximation, we have theoretically investigated the exciton-related Raman scattering, interband absorption and photoluminescence in colloidal CdSe/CdS core/shell quantum dots ensemble. The interband optical absorption and photoluminescence spectra have been revealed for CdSe/CdS quantum dots, taking into account the size dispersion of the ensemble. Numerical calculation of the differential cross section has been presented for the exciton-related Stokes–Raman scattering in CdSe/CdS quantum dots ensemble with different mean sizes.

## 1. Introduction

In recent years, interest in quantum dots (QDs) has increased, because of their wide range of uses in various devices, from photovoltaic cells to QD batteries. The problem for most of the QDs is that manufacturing methods have been costly, requiring high vacuum and high temperature. Colloidal QDs are an exception to that rule and their manufacturing processes use low-temperature, large area, solution-based methods. Colloidal semiconductor QDs’ low-cost manufacturing provides an opportunity for industrial use. Some applications of colloidal QDs include, but are not limited to, photodiodes and photovoltaics, photoconductors, electroluminescent devices, advanced batteries, etc. [1,2,3,4,5]. Core-shell colloidal QDs are at the bleeding edge of the hot research topics, due to their excellent properties, such as versatility, tunability, and stability. They have attracted considerable interest attributed to their dramatically tunable physical properties. Core-shell structures have been generally defined as structures consisting of a core (inner material) and a shell (outer layer material) [6,7,8,9]. In general, many considerable efforts on core-shell materials have been reported, such as nanoparticles, spheres, nanowires, nanorods, nanotubes, nanobelts, nanofibers, nanoplates, nanosheets, cubes, flowers [10,11,12,13,14,15], etc. The main advantages of using core shell QDs are the following: core shell structures can protect the core from the effect of environmental changes; intensify or bring new physical capabilities; limit volume expansion, and as a result, maintaining structural integrity; keep the core from forming into large particles; and percolate ions or molecules onto the core selectively.

Recently, one of the most interesting materials for the manufacturing of core/shell QDs is CdSe/CdS. They have been of particular interest for their unique optical properties, which have been thoroughly investigated [16,17,18]. These particles have exhibited high PL quantum yields and excellent photostability, which has made them particularly interesting [19,20]. Most CdSe/CdS core/shell particles are synthesized at high temperatures and have a wurtzite crystal structure, which is the most stable form [21,22,23].

One of the powerful methods for the investigation of QDs is Raman spectroscopy, which has been used in a considerable range of research activity. In solid state physics, the position, shape and intensity of the Raman peak can give insight into the lattice dynamics and the electronic configuration of the material [24]. On the other hand, the calculation of the differential cross-section (DCS) of Raman scattering remains a rather interesting and fundamental issue to achieve a better understanding of semiconductor nanostructures. Raman scattering of an exciton in a quantum dot has been investigated in [25,26,27,28,29].

In [26], a theoretical calculation of the DCS is presented for the exciton-mediated Stokes–Raman scattering in CdS quantum dot within the framework of the effective mass approximation at T=0 K. Numerical calculations reveal that the excitonic effects cause blue shifts in Raman spectroscopy and the magnitude of the Raman shift depends on the quantum confinement, the Coulomb interaction, and the incident photon energy.

In another work, the authors have investigated the thermal properties of colloidal CdSe/CdS QDs associated with the additional epitaxial strain at the core/shell interface. They observed the temperature-dependent behavior of the longitudinal optical phonon frequencies of these QDs by Raman spectroscopy over a temperature range of 7–300 K [27].

Besides the Raman spectroscopy, one of the additional widely used investigation methods of semiconductor QDs is photoluminescence (PL) spectroscopy. There are number of works devoted to the investigation of PL properties of core/shell structures [30,31,32,33,34]. For example, in [30], the authors analyzed the excitation energy dependence of the PL quantum yields and decay kinetics and the circular dichroism spectra of CdSe/CdS core/shell QDs with different thicknesses of the surface passivation shell.

Thus, the investigation of Raman and PL spectra for CdSe/CdS core/shell colloidal QDs is a problem of the current interest problem. In this paper, the exciton related Raman scattering, optical absorption and photoluminescence spectrum in colloidal CdSe/CdS core/shell quantum dots with different sizes have been theoretically investigated in the framework of the effective mass approximation. The exciton states in colloidal CdSe/CdS core/shell QD have been calculated using numerical discretization method. The paper is organized as follows: the chosen model of the colloidal CdSe/CdS core/shell QD has been discussed in Section 2; interband absorption, photoluminescence and the formula of the Raman scattering DCS have been presented in Section 3, the calculation results and discussion have been presented in Section 4, and the conclusions have been shown in Section 5.

## 2. Exciton States

Before proceeding to the theoretical calculation part, let’s discuss the structure and corresponding model of the observed colloidal CdSe/CdS core/shell QD. In the schematic plot of the above-mentioned QD with the corresponding energy band diagram has been presented. The real material parameters for these structures have been taken from the book [35].

**Figure 1:**
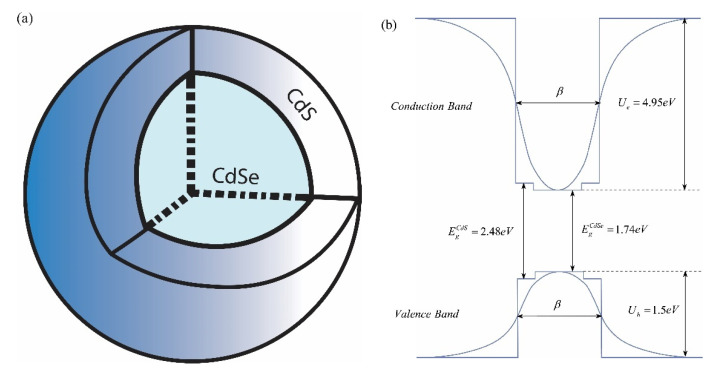
The schematic plot of CdSe/CdS core/shell QD (**a**) with corresponding energy band structure (**b**).

As can be seen from Figure 1b, the real band diagram can be modeled and fitted by the smoother confinement potential, if we take into account that during the growth process, due to the diffusion, the blurring of the sharp boundaries occurs. For the aforementioned reasons, the modified Pöschl–Teller potential (MPTP) has been chosen as the model of the confinement potential for further calculations. There are many works devoted to the modeling of quantum nanostructures, where the MPTP has been applied [36,37,38].

Within the framework of effective-mass approximation, the Hamiltonian of the exciton system for the radial part in spherical coordinates can be written in the form:

(1) $$\hat{H}=-\frac{\hbar^{2}}{2m_{e}^{*}}\Delta_{e}-\frac{\hbar^{2}}{2m_{h}^{*}}\Delta_{h}-\frac{e^{2}}{\varepsilon \left|{\vec{r}}_{e}-{\vec{r}}_{h}\right|}+U_{conf}\left({\vec{r}}_{e}\right)+U_{conf}\left({\vec{r}}_{h}\right)$$

in which $m_{e}^{*}$ and $m_{h}^{*}$ are the effective masses of electron and hole respectively, e is the electron charge magnitude, ε is the dielectric constant, $U_{conf}\left(\vec{r}\right)$ is the confinement potential. It should be noted that the angular parts are irrelevant for the discussed problem, thus the ground states of the magnetic and orbital quantum numbers have been discussed. The analytic expression of the modeling potential has the following form:

(2) $$U_{conf}\left({\vec{r}}_{e\left(h\right)}\right)=U_{e\left(h\right)}-\frac{U_{e\left(h\right)}}{\cosh^{2}\left(r_{e\left(h\right)}/\beta \right)}$$

where $U_{e\left(h\right)}$ and β are respectively the depth and half-width of the MPTP. The numerical discretization method, in other words, the finite element method, has been performed for the realization of the numerical calculations. As the standard procedure, first the discrete representation of the considered region has been done; that is, the mesh structure of the region has been constructed with appropriate accuracy. The region is two dimensional, namely $r_{e}$ and $r_{h}$ radial coordinates are considered, since the angular coordinates are irrelevant, both for electron and hole. As the next step, partial differential time-independent Schrödinger equation is transformed to a system of algebraic equations taking into account Dirichlet boundary conditions. Finally, the set of algebraic equations is solved.

**Figure 2:**
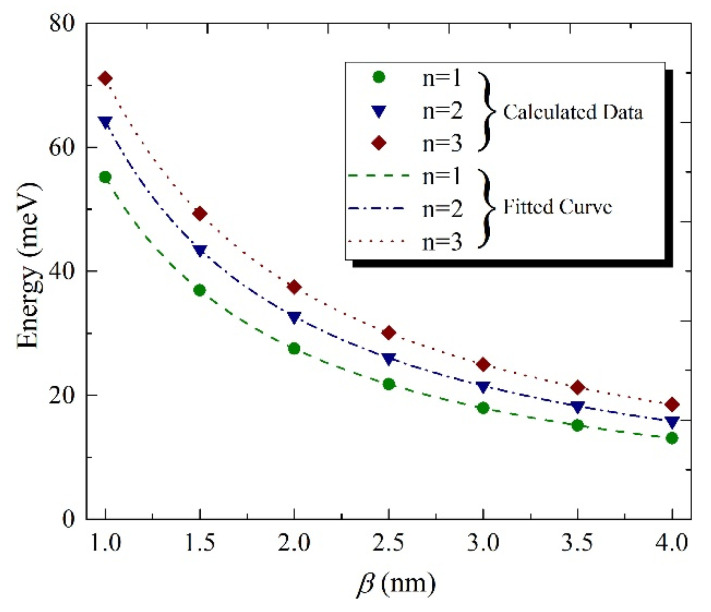
The dependence of the exciton energy on the halfwidth for the first three levels. Dots correspond to the values obtained by the numerical calculations. The lines correspond to the fitted curve which has been fitted to the calculated data points. The following model for the curve was selected: $E_{n}=A_{n}+\frac{B_{n}}{\beta }+\frac{C_{n}}{\beta^{2}}$.

The numerical calculations have shown that the dependence of the energy spectra on half-width β can be fitted as:

(3) $$E_{n}=A_{n}+\frac{B_{n}}{\beta }+\frac{C_{n}}{\beta^{2}}$$

where the values of parameters $A_{n}$, $B_{n}$ and $C_{n}$ can be found by the direct numerical calculations and n is the principal quantum number. The dependence of the exciton energy on the halfwidth for the first three levels are plotted in Figure 2. From the figure, it is clearly visible that the energy dependence fits with high accuracy to the model (3). The numerical values for the above-mentioned parameters for the first three levels have been presented in the Table 1. These values will be used in the next section for the calculation of DCS.

## 3. Optical Properties

In this section, the interband absorption of the incident light, photoluminescence and Raman spectra of CdSe/CdS QD have been presented. The light absorption coefficient with the account of spectral lines broadening has the following form:

(4) $$\alpha \left(\hbar \omega ,\beta \right)=\alpha_{0}{\sum_{\nu_{e},\nu_{h}}\left|\int \Psi_{exc}\left({\vec{r}}_{e},{\vec{r}}_{h}\right)d{\vec{r}}_{e}d{\vec{r}}_{h}\right|}^{2}\frac{\Gamma_{0}}{\left({\left(\hbar \omega_{e,h}-E_{g}^{L}-E_{e}\left(\beta \right)-E_{h}\left(\beta \right)\right)}^{2}+\Gamma_{0}^{2}\right)}$$

where $E_{g}$ is the band gap of the semiconductor, $\alpha_{0}$ is a quantity proportional to the square of the dipole moment’s matrix element taken over the Bloch functions, $\Gamma_{0}$ is the width of Lorentzian parameter and $\psi_{exc}\left({\vec{r}}_{e},{\vec{r}}_{h}\right)$ is exciton wave function [39,40]. With the help of $\Gamma_{0}$ parameter, the broadening of the absorption lines has been accounted for. The broadening of the spectral lines has two different realization mechanisms: homogeneous and inhomogeneous. We will consider the homogeneous broadening with empirical method, taking the parameter value from the experiment. As for the inhomogeneous broadening, caused by the random size distribution of the QDs ensemble, it has been calculated, taking into account size dispersion. We will discuss the case where the geometrical sizes have the Gaussian distribution with the variation not exceeding 10% from the mean value.

Taking into account the aforementioned, the average light absorption coefficient of an ensemble of core/shell QDs with a size distribution function F(β) can be expressed as:

(5) 〈α(ℏω,β)〉=∫F(β)α(ℏω,β)dβ

Using the absorption spectra, it is possible to calculate photoluminescence (PL) spectra for the core/shell QD, with the Roosbroeck–Shockley relation [41,42]:

(6) $$R\left(\hbar \omega ,\beta \right)=R_{0}\hbar \omega \;\alpha \left(\hbar \omega ,\beta \right)\frac{f_{c}\left(1-f_{v}\right)}{f_{v}-f_{c}}$$

where $R_{0}$ is proportional to the $\alpha_{0}$, $f_{c}$ and $1-f_{v}$ are probabilities of the conduction band states being occupied and the valance band states being empty, respectively. The average PL spectra 〈R(ℏω,β)〉 will be calculated with the same procedure as for absorption spectra:

(7) 〈R(ℏω,β)〉=∫F(β)R(ℏω,β)dβ

As for the calculation of the exciton-related Stokes–Raman scattering DCS in a volume V, per unit solid angle dΩ, the expression obtained by the third-order perturbation theory has the following form:

(8) $$\frac{d^{2}\sigma }{d\Omega d\omega_{s}}=\frac{V^{2}\omega_{s}^{2}\eta \left(\omega_{s}\right)}{8\pi^{3}c^{4}\eta \left(\omega_{l}\right)}W\left(\omega_{s},{\vec{e}}_{s}\right)$$

Here, c is the light velocity, $\omega_{l}$ is the frequency of incident light, $\omega_{s}$ represents the frequency of the scattered light, $\eta \left(\omega_{l}\right)$ and $\eta \left(\omega_{s}\right)$ are the refractive indices for the incident and scattered light, respectively, ${\vec{e}}_{s}$ is the polarization vector [43,44,45]. $W\left(\omega_{s},{\vec{e}}_{s}\right)$ is the transition rate, which can be calculated by:

(9) $$W\left(\omega_{s},{\vec{e}}_{s}\right)=\frac{2\pi }{\hbar }\sum_{f}{\left|M_{e}+M_{h}\right|}^{2}\delta \left(E_{f}-E_{i}\right)$$

where M and $\delta \left(E_{f}-E_{i}\right)$ are defined by the following expressions:

(10) $$M_{j}=\sum_{a}\frac{\langle f\left|H_{js}\right|a\rangle \langle a\left|H_{jl}\right|i\rangle }{E_{i}-E_{a}+i\Gamma_{a}},\;\;\;\;j=e,h$$

and

(11) $$\delta \left(E_{f}-E_{i}\right)=\frac{\Gamma_{f}}{\pi \left\{{\left(E_{f}-E_{i}\right)}^{2}+\Gamma_{f}^{2}\right\}}$$

Let’s note that |i〉, |a〉 and |f〉 describe the initial, intermediate and final states in the system with energies $E_{i}$, $E_{a}$ and $E_{f}$, respectively (see Table 1) and $\Gamma_{f}$ is the life-time width. In the dipole approximation, the interaction with the incident and secondary radiation fields can be written by the Hamiltonian operators:

(12) $${\hat{H}}_{jl}=\frac{\left|e\right|}{m_{e}}\sqrt{\frac{2\pi \hbar }{V\omega_{l}}}\left({\vec{e}}_{jl}\cdot {\vec{p}}_{j}\right),\;\;\;{\vec{p}}_{j}=-i\hbar {\vec{\nabla }}_{j}$$

(13) $${\hat{H}}_{js}=\frac{\left|e\right|}{m_{j}^{*}}\sqrt{\frac{2\pi \hbar }{V\omega_{s}}}\left({\vec{e}}_{js}\cdot {\vec{p}}_{j}\right)$$

where, $m_{e}$ is the free electron mass, ${\vec{e}}_{jl}$ and ${\vec{e}}_{js}$ are the unit polarization vectors for the incident and secondary radiations. The matrix elements of DCS have been calculated using numerical values for the energy spectra and wave functions obtained in Section 2. Finally, for the averaging DCS spectra, we will take into account size distribution function F(β):

(14) $$\langle \frac{d^{2}\sigma }{d\Omega d\omega_{s}}\rangle =\int F\left(\beta \right)\frac{d^{2}\sigma }{d\Omega d\omega_{s}}d\beta$$

## 4. Results and Discussion

Let us proceed to the discussion of the obtained results. The material parameters that have been used during the calculations are the following: $m_{e}^{*}\left(CdS\right)=0.15m_{0}$, $m_{h}^{*}\left(CdS\right)=0.7m_{0}$, ε(CdS)=8.9, $m_{e}^{*}\left(CdSe\right)=0.149m_{0}$, $m_{h}^{*}\left(CdSe\right)=0.45m_{0}$, ε(CdSe)=8.34, where $m_{0}$ is the free electron mass [35]. For both materials, the wurtzite structure has been chosen. The homogeneous broadening linewidth is taken to be $\Gamma_{0}=45÷55\;\mathrm{meV}$ CdSe for different sized QDs [46]. The parameter for CdSe was taken as the main localization area for the exciton is the core layer.

**Figure 3:**
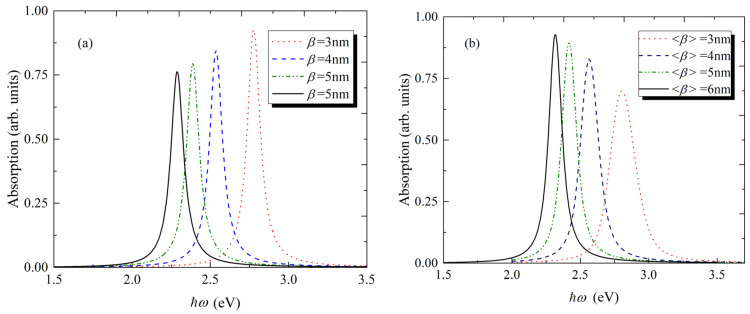
The dependence of the absorption spectra on the energy of incident light for different sizes of CdSe/CdS core/shell QD (**a**) for single QD, (**b**) for the ensemble of QDs.

In Figure 3a, the dependence of the absorption spectra for the single QD on the energy of incident light for different sizes CdSe/CdS core/shell QD have been presented. As we can see from the figure, with the decrease of the QD size, the absorption peak has a blue shift. The shift has asymmetric character, and moreover, for the smaller sizes, the shift is more pronounced. This is the expression of the size quantization: the QDs with smaller sizes have higher exciton energy. There is also a small difference in between-peaks linewidth, due to the dependence of the $\Gamma_{0}$ parameter on the geometrical sizes of QD. In Figure 3b, the same dependence is presented for the QDs ensemble. In the figure, the mean values of β parameter are presented for different ensembles. Compared to the single QD the halfwidth of spectra has increased. For example, for the single QD with β=3 nm, the absorption halfwidth is ΔΓ≃0.1 eV, compared to ΔΓ≃0.21 eV for the ensemble, when the mean value of the QDs sizes is <β>=3 nm. The broadening can be explained by the increase of effective cross section of the absorption surface in ensemble in comparison to single QD. In this case, the number of possible absorptions of incident photons will be more than for single QD.

Despite the maximum of the peak intensity being higher for the QDs of smaller size, in the ensemble, the opposite behavior is observed: the peak intensity decreases with the decreases of QDs size. The same explanation can be applied for this effect: the effective surface that will absorb incident light is larger for the ensemble of QDs with large average size value, when the concertation of the QDs is equal in the unit volume.

**Figure 4:**
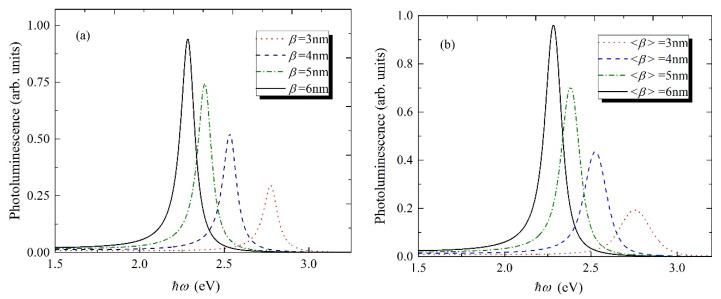
The dependence of the PL spectra light energy for different sizes of CdSe/CdS core/shell QD (**a**) for single QD, (**b**) for the ensemble of QDs.

For the calculation of the PL spectra for both single QD and QDs ensemble, the Equations (6) and (7) have been used respectively. The PL spectra dependence on light energy has been presented in Figure 4a,b. The peak positions in both figures are the same, however, the spectra lines for the ensemble of QDs have observable broadening. For example, for the single QD with β=3 nm the PL halfwidth is ΔΓ≃0.1 eV, compared to ΔΓ≃0.21 eV for the ensemble. Thus, the homogenous and inhomogeneous broadenings approximately have the same contribution to the spectral line expansion for both PL and absorption spectra. Note that dependences have been calculated and plotted for the 4.2 K temperature.

For the estimation of the radiative lifetime of exciton states in CdSe/CdS QD, we use the formula obtained in [47]:

(15) $$\tau_{exc}=\frac{2\pi \varepsilon_{0}m_{0}c^{3}\hbar^{2}}{\sqrt{\varepsilon }e^{2}E_{exc}^{2}f}$$

where ε is dielectric constant, $\varepsilon_{0}$ is vacuum permittivity, $E_{exc}$ is the energy of an exciton, and f is the oscillator strength, which is defined by the formula:

(16) $$f=\frac{E_{P}}{E_{exc}}{\left|\int \Psi_{exc}\left({\vec{r}}_{e},{\vec{r}}_{h}\right)d{\vec{r}}_{e}d{\vec{r}}_{h}\right|}^{2}$$

where $E_{P}$ is the Kane energy and it is $E_{P}=21\;\mathrm{eV}$ for CdSe [48]. Note, that for the estimation of the radiative lifetime, we have neglected interaction between exciton and phonons. The estimated radiative lifetimes for different QD sizes are presented in the Table 2. The characteristic times are in the picoseconds range. As it can be seen from Table 2, with the increase of QD size, the radiative lifetime increases correspondingly. The increase of the radiative lifetime can be explained with the following: with the increase of the QD size, the exciton energy and the overlap integral in the oscillator strength decrease, as the size quantization effect becomes weaker. Because the radiative lifetime is inversely proportional to the above-mentioned quantities, with the increase of the QD size, the lifetime will increase.

**Figure 5:**
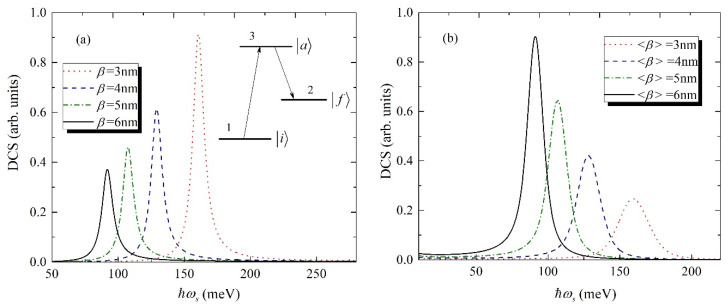
Exciton-related Raman DCS as a function of secondary-radiation photon energy for different sizes of CdSe/CdS core/shell QD, (**a**) for single QD, inset: three-level scheme, (**b**) for the ensemble of QDs.

Finally, the exciton-related Raman DCS as the function of secondary-radiation photon energy for different sizes of CdSe/CdS core/shell QD has been considered. The DCS of Stokes Raman scattering for a three-level system is calculated for a single CdSe/CdS QD using Equation (8). As the initial state, the ground state of the exciton has been chosen |i〉, we consider Raman excitations from this state. The incident photon energy is $\hbar \omega_{l}$, while the energy of scattered light is $\hbar \omega_{s}$. The first transition occurs between the ground state with the energy $E_{i}$ to the intermediate state |a〉 with the energy of $E_{a}$, which is the second excited state. The final transition occurs between the intermediate state |a〉 and the final state |f〉 with the energy $E_{f}$, which is the first excited state. The energy of the secondary radiation $\hbar \omega_{s}$ is the energy emitted from the abovementioned final transition. It is obvious, that as the intermediate state |a〉, all excited states starting with the second can be considered. However, for simplicity, we will discuss the case, when the intermediate state is second excited state. This approximation is justified by the relatively small contribution of the transitions from the higher excites states. The three-level transition processes (1→3→2) with the results for the Raman DCS have been presented in Figure 5a. The lifetimes widths of the final and intermediate states are chosen as follows: $\Gamma_{f}=\Gamma_{a}=50\;\mathrm{meV}$ [46]. It should be mentioned that these empirically measured lifetimes widths are different from radiative lifetime, because the exciton phonon interaction is considered.

Finally, the DCS of Stokes–Raman scattering for QDs ensemble is calculated using the Equation (14), taking into account the size dispersion of the QDs. The main characteristics are listed as follows. First, the intensities of the Raman DCS increase with the decrease of the single QD size, but for the ensemble, it has opposite behavior. Secondly, the broadening of the peaks is more pronounced for the ensemble of CdSe/CdS QDs, because the effective cross section of the active area is more for the ensemble; as a result, the number of Raman scattered photons is higher.

## 5. Conclusions

In the present article, we have theoretically studied the exciton related Stokes–Raman scattering, interband absorption and photoluminescence in colloidal CdSe/CdS core/shell quantum dots ensemble. It has been shown that, with the decrease of the QD’s size, the absorption peak has a blue shift, and it is more pronounced for the QDs with smaller sizes. With the consideration of size dispersion, the linewidth of absorption spectral lines become broader, and also the peak intensity decreases with the decreases of mean QD size in ensemble. For PL spectra, the same pattern is true. Moreover, the conclusion from the abovementioned is that the homogenous and inhomogeneous broadenings approximately have the same contribution to the spectral line expansion for both PL and absorption spectra of CdSe/CdS QDs ensemble.

The estimation of radiative lifetime shows that the exciton characteristic lifespan time lies in the picosecond region. Moreover, with the increase of the QD sizes, the exciton lifetime approaches to nanosecond. We have also investigated the Stokes–Raman scattering of an exciton in a CdSe/CdS QDs. The results are presented as a function of the scattered photon energy. The intensities of the Raman DCS increase with the decrease of the single QD size, but for the ensemble, has opposite behavior. In addition, the broadening of the DCS peaks is more pronounced for the ensemble of CdSe/CdS QDs. Presented results will be useful for identifying the exciton-related Raman scattering in semiconductor quantum nanosystems.

## Figures and Tables

**Table 1:** This dimensionless numerical values for the parameters $A_{n}$, $B_{n}$ and $C_{n}$. All of the energies have been presented in Rydberg energy $E_{R}$ and all lengths in Bohr radius $a_{B}$. For the Rydberg, energy is equal to $E_{R}=18.85\;\mathrm{meV}$, and Bohr radius is $a_{B}=2.98\;\mathrm{nm}$.

n	$A_{n}/E_{R}$	$\left(B_{n}\;a_{B}\right)/E_{R}$	$\left(C_{n}\;a_{B}^{2}\right)/E_{R}$
1 (i)	−1.816	60.339	−3.338
2 (f)	−1.809	71.940	−5.873
3 (a)	−1.886	84.390	−11.360

**Table 2:** Dimensionless numerical values for the parameters $A_{n}$, $B_{n}$ and $C_{n}$. All of the energies have been presented in Rydberg energy $E_{R}$ and all lengths in Bohr radius $a_{B}$. For the Rydberg energy is equal to $E_{R}=18.85\;\mathrm{meV}$, and Bohr radius is $a_{B}=2.98\;\mathrm{nm}$.

	β=3 nm	β=4nm	β=5 nm	β=6 nm
$\tau_{exc}$, ps	367	487	610	736

## References

[1] Kagan C.R., Lifshitz E., Sargent E.H., Talapin D.V. (2016). Building devices from colloidal quantum dots. Science.

[2] Lu W., Guo X., Luo Y., Li Q., Zhu R., Pang H. (2019). Core-shell materials for advanced batteries. Chem. Eng. J..

[3] Clifford J.P., Konstantatos G., Johnston K.W., Hoogland S., Levina L., Sargent E.H. (2008). Fast, sensitive and spectrally tuneable colloidal-quantum-dot photodetectors. Nat. Nanotechnol..

[4] Konstantatos G., Howard I.S., Fischer A., Hoogland S., Clifford J.P., Klem E.J.D., Levina L., Sargent E.H. (2006). Ultrasensitive solution-cast quantum dot photodetectors. Nat. Cell Biol..

[5] Chen O., Zhao J., Chauhan V.P., Cui J., Wong C., Harris D.K., Wei H., Han H.-S., Fukumura D., Jain R.K. (2013). Compact high-quality CdSe–CdS core–shell nanocrystals with narrow emission linewidths and suppressed blinking. Nat. Mater..

[6] Vasudevan D., Gaddam R.R., Trinchi A., Cole I. (2015). Core–shell quantum dots: Properties and applications. J. Alloys Compd..

[7] Selopal G.S., Zhao H., Wang Z.M., Rosei F. (2020). Core/shell quantum dots solar cells. Adv. Funct. Mater..

[8] Yuan X., Zheng J., Zeng R., Jing P., Ji W., Zhao J., Yang W., Li H. (2014). Thermal stability of Mn2+ion luminescence in Mn-doped core–shell quantum dots. Nanoscale.

[9] Zeng Z., Garoufalis C.S., Terzis A.F., Baskoutas S. (2013). Linear and nonlinear optical properties of ZnO/ZnS and ZnS/ZnO core shell quantum dots: Effects of shell thickness, impurity, and dielectric environment. J. Appl. Phys..

[10] Zeiri N., Naifar A., Nasrallah S.A.-B., Said M. (2019). Third nonlinear optical susceptibility of CdS/ZnS core-shell spherical quantum dots for optoelectronic devices. Optik.

[11] Loitsch B., Winnerl J., Grimaldi G., Wierzbowski J., Rudolph D., Morkotter S., Finley J.J. (2015). Crystal phase quantum dots in the ultrathin core of GaAs–AlGaAs core–shell nanowires. Nano Lett..

[12] Hayrapetyan D.B., Kazaryan E.M., Petrosyan L.S., Sarkisyan H.A. (2015). Core/shell/shell spherical quantum dot with Kratzer confining potential: Impurity states and electrostatic multipoles. Phys. E Low-Dimens. Syst. Nanostruct..

[13] Jiang H., Chen Y., Li L., Liu H., Ren C., Liu X., Tian G. (2020). Hierarchical ZnO nanorod/ZnFe2O4 nanosheet core/shell nanoarray decorated with PbS quantum dots for efficient photoelectrochemical water splitting. J. Alloys Compd..

[14] Kayaci F., Vempati S., Ozgit-Akgun C., Donmez I., Biyikli N., Uyar T. (2015). Transformation of polymer-ZnO core–shell nano-fibers into ZnO hollow nanofibers: Intrinsic defect reorganization in ZnO and its influence on the photocatalysis. Appl. Catal. B Environ..

[15] Wang M., Peng Z., Qian J., Li H., Zhao Z., Fu X. (2018). Highly efficient solar-driven photocatalytic degradation on environmental pollutants over a novel C fibers MoSe2 nanoplates core-shell composite. J. Hazard. Mater..

[16] Lin C., Gong K., Kelley D.F., Kelley A.M. (2015). Electron–Phonon Coupling in CdSe/CdS Core/Shell Quantum Dots. ACS Nano.

[17] Kong D., Jia Y., Ren Y., Xie Z., Wu K., Lian T. (2018). Shell-thickness-dependent biexciton lifetime in type I and quasi-type II CdSe@ CdS core/shell quantum dots. J. Phys. Chem. C.

[18] Gong K., Kelley D.F. (2014). A predictive model of shell morphology in CdSe/CdS core/shell quantum dots. J. Chem. Phys..

[19] Greytak A.B., Allen P.M., Liu W., Zhao J., Young E.R., Popović Z., Walker B.J., Nocera D.G., Bawendi M.G. (2012). Alternating layer addition approach to CdSe/CdS core/shell quantum dots with near-unity quantum yield and high on-time fractions. Chem. Sci..

[20] Hu Z., Liu S., Qin H., Zhou J., Peng X. (2020). Oxygen Stabilizes Photoluminescence of CdSe/CdS Core/Shell Quantum Dots via Deionization. J. Am. Chem. Soc..

[21] Peng X., Schlamp M.C., Kadavanich A.V., Alivisatos A.P. (1997). Epitaxial Growth of Highly Luminescent CdSe/CdS Core/Shell Nanocrystals with Photostability and Electronic Accessibility. J. Am. Chem. Soc..

[22] Luo Y., Wang L.-W. (2010). Electronic Structures of the CdSe/CdS Core−Shell Nanorods. ACS Nano.

[23] Tan R., Yuan Y., Nagaoka Y., Eggert D., Wang X., Thota S., Chen O. (2017). Monodisperse hexagonal pyramidal and bipyramidal wurtzite CdSe-CdS core–shell nanocrystals. Chem. Mater..

[24] Ferrari A.C. (2007). Raman spectroscopy of graphene and graphite: Disorder, electron–phonon coupling, doping and nonadiabatic effects. Solid State Commun..

[25] Xie W. (2013). Raman scattering of an exciton in a quantum dot. Phys. B Condens. Matter.

[26] Guo X., Liu C. (2017). Exciton-mediated Raman scattering in CdS quantum dot. Phys. E Low Dimens. Syst. Nanostruct..

[27] Badlyan N.M., Biermann A., Aubert T., Hens Z., Maultzsch J. (2019). Thermal expansion of colloidal CdSe/CdS core/shell quantum dots. Phys. Rev. B.

[28] Xie W. (2012). Raman scattering of a donor impurity in a quantum ring. Chem. Phys..

[29] Lu L., Xu X.-L., Liang W.-T., Lu H.-F. (2007). Raman analysis of CdSe/CdS core–shell quantum dots with different CdS shell thickness. J. Phys. Condens. Matter.

[30] Martynenko I.V., Baimuratov A.S., Osipova V.A., Kuznetsova V.A., Purcell-Milton F., Rukhlenko I.D., Baranov A.V. (2018). Ex-citation energy dependence of the photoluminescence quantum yield of core/shell CdSe/CdS quantum dots and correlation with circular dichroism. Chem. Mater..

[31] Stroyuk O., Raevskaya A., Gaponik N., Selyshchev O., Dzhagan V., Schulze S., Zahn D.R. (2018). Origin of the broadband pho-toluminescence of pristine and Cu+/Ag+-doped ultrasmall CdS and CdSe/CdS quantum dots. J. Phys. Chem. C.

[32] Dunlap M.K., Ryan D.P., Goodwin P.M., Werner J.H., Majumder S., Hollingsworth J.A., Gelfand M.P., Van Orden A. (2020). Super-resolution photoluminescence lifetime and intensity mapping of interacting CdSe/CdS quantum dots. Appl. Phys. Lett..

[33] Purcell-Milton F., Visheratina A.K., Kuznetsova V.A., Ryan A., Orlova A.O., Gun’Ko Y.K. (2017). Impact of Shell Thickness on Photoluminescence and Optical Activity in Chiral CdSe/CdS Core/Shell Quantum Dots. ACS Nano.

[34] Luo C.-L., Yang R.-X., Yan W.-G., Chen C.-M., Liu S.-Y., Zhao S.-J., Ge W.-Q., Liu Z.-F., Jia G.-Z. (2019). Surface Plasmon-Enhanced Luminescence of CdSe/CdS Quantum Dots Film Based on Au Nanoshell Arrays. Materials.

[35] Poerschke R., Madelung O. (1992). Semiconductors: Other than Group IV Elements and 2I-V Compounds.

[36] Parang Z., Keshavarz A., Zamani N. (2014). Optimization of optical absorption coefficient in double modified Pöschl–Teller quantum wells. J. Comput. Electron..

[37] Hayrapetyan D.B., Kazaryan E.M., Tevosyan H.K. (2013). Optical properties of spherical quantum dot with modified Pöschl–Teller potential. Superlattices Microstruct..

[38] Karimi M., Rezaei G. (2011). Effects of external electric and magnetic fields on the linear and nonlinear intersubband optical properties of finite semi-parabolic quantum dots. Phys. B Condens. Matter.

[39] Efros A.L., Efros A.L. (1982). Interband absorption of light in a semiconductor sphere. Semiconductors.

[40] Ramaniah L.M., Nair S.V. (1993). Optical absorption in semiconductor quantum dots: A tight-binding approach. Phys. Rev. B.

[41] Van Roosbroeck W., Shockley W. (1954). Photon-Radiative Recombination of Electrons and Holes in Germanium. Phys. Rev..

[42] Bhattacharya R., Pal B., Bansal B. (2012). On conversion of luminescence into absorption and the van Roosbroeck-Shockley relation. Appl. Phys. Lett..

[43] Chamberlain M.P., Trallero-Giner C., Cardona M. (1995). Theory of one-phonon Raman scattering in semiconductor microcrys-tallites. Phys. Rev. B.

[44] Trallero-Giner C., Debernardi A., Cardona M., Menendez-Proupin E., Ekimov A.I. (1998). Optical vibrons in CdSe dots and dis-persion relation of the bulk material. Phys. Rev. B.

[45] Trallero-Giner C., Cardona M. (1997). Vibrational Resonant Raman Scattering in Spherical Quantum Dots: Exciton Effects. Phys. Status Solidi B.

[46] Gellen T.A., Lem J., Turner D.B. (2017). Probing Homogeneous Line Broadening in CdSe Nanocrystals Using Multidimensional Electronic Spectroscopy. Nano Lett..

[47] Fonoberov V.A., Balandin A.A. (2004). Origin of ultraviolet photoluminescence in ZnO quantum dots: Confined excitons versus surface-bound impurity exciton complexes. Appl. Phys. Lett..

[48] Gerdova I., Haché A. (2005). Third-order non-linear spectroscopy of CdSe and CdSe/ZnS core shell quantum dots. Opt. Commun..

